# A juvenile locomotor program promotes vocal learning in zebra finches

**DOI:** 10.1038/s42003-022-03533-3

**Published:** 2022-06-10

**Authors:** Wan-chun Liu, Michelle Landstrom, MaKenna Cealie, Iona MacKillop

**Affiliations:** Department of Psychological and Brain Sciences, Colgate University, Hamilton, NY USA

**Keywords:** Birdsong, Social behaviour

## Abstract

The evolution and development of complex, learned motor skills are thought to be closely associated with other locomotor movement and cognitive functions. However, it remains largely unknown how different neuromuscular programs may interconnect during the protracted developmental process. Here we use a songbird to examine the behavioral and neural substrates between the development of locomotor movement and vocal-motor learning. Juvenile songbirds escalate their locomotor activity during the sensitive period for vocal learning, followed by a surge of vocal practice. Individual variability of locomotor production is positively correlated with precision of tutor imitation and duration of multi-syllable sequences. Manipulation of juvenile locomotion significantly impacts the precision of vocal imitation and neural plasticity. The locomotor program developed during the sensitive period of vocal learning may enrich the neural substrates that promote the subsequent development of vocal learning.

## Abstract (web summary)

Increased movement is associated with improved vocal activity in young zebra finches, while reducing locomotion leads to poor vocal learning. These results suggest that exercise in juvenile birds may actually promote their ability to learn and produce songs.

## Introduction

The development of complex sensorimotor-learning skills is closely associated with the development of locomotor and cognitive functions^1–3^. In humans, for example, the development of language or speech learning is thought to be part of developing general motor systems that involve broader sensorimotor integration and neuromuscular control. Early milestones of language development are closely linked to developmental transitions of other locomotor skills, such as an infant learning to walk from crawling, and the emergence of early gesture or rhythmic movement^4–6^. Moreover, in children with autism, early locomotor deficits often precede and predict the later emergence of language or cognitive impairments^7–10^. This close association between developing locomotor and sensorimotor learning could be due to a more general, shared program (such as intrinsic motivation state) that develops and operates independently on different neuromuscular systems. Alternatively, early locomotor activity and its developmental transitions may activate a wide range of neural plasticity in neuromuscular systems (for example, gene expression, neurogenesis, or synaptogenesis), which may foster the subsequent development of sensorimotor learning or cognitive functions. This hypothesis is supported by a number of studies in which a training intervention of locomotor activity in children can improve speech or other cognitive development^2^. Early locomotor activity may thus not be required, but could promote the later development of other sensorimotor learning skills.

In this study, we investigate whether there is a developmental association between locomotor movement and vocal learning in songbirds. Songbirds have great potential as an animal model to examine and manipulate the behavioral and neuronal substrates that underlie the close association between developing fine motor-learning skills and developing locomotor movement. When a young songbird first comes out of the nest and transitions from perching to flight, the motor practice of flight movement requires substantial reconfiguration and integration of respiratory and rhythmic control, sensory perception, and muscular coordination^11–13^. In the meantime, they develop the learned song during a limited sensitive period^14^. It is unknown whether these different sensorimotor systems in songbirds are associated or interact with each other during development. Interestingly, in songbirds, the locomotor and vocal learning systems are under the control of two closely adjacent cortico-basal ganglia circuits, suggesting possible evolutionary or developmental connections^15^.

To test this hypothesis, we use a songbird, the zebra finch (*Taeniopygia gutatta*) as an animal model to quantitatively track the developmental trajectory of juvenile locomotor movement and its association with the development of song learning, and whether the developing locomotor activity may affect the neural plasticity of the developing forebrain song circuit. Here we provide the experimental evidence that juvenile zebra finches develop a locomotor program that precedes the vocal-motor program and surges during the sensitive period for song learning. This juvenile locomotor program is closely tied to the subsequent development of song learning, and affects the neurogenesis of the developing song circuits.

## Results

**Fig. 1:**
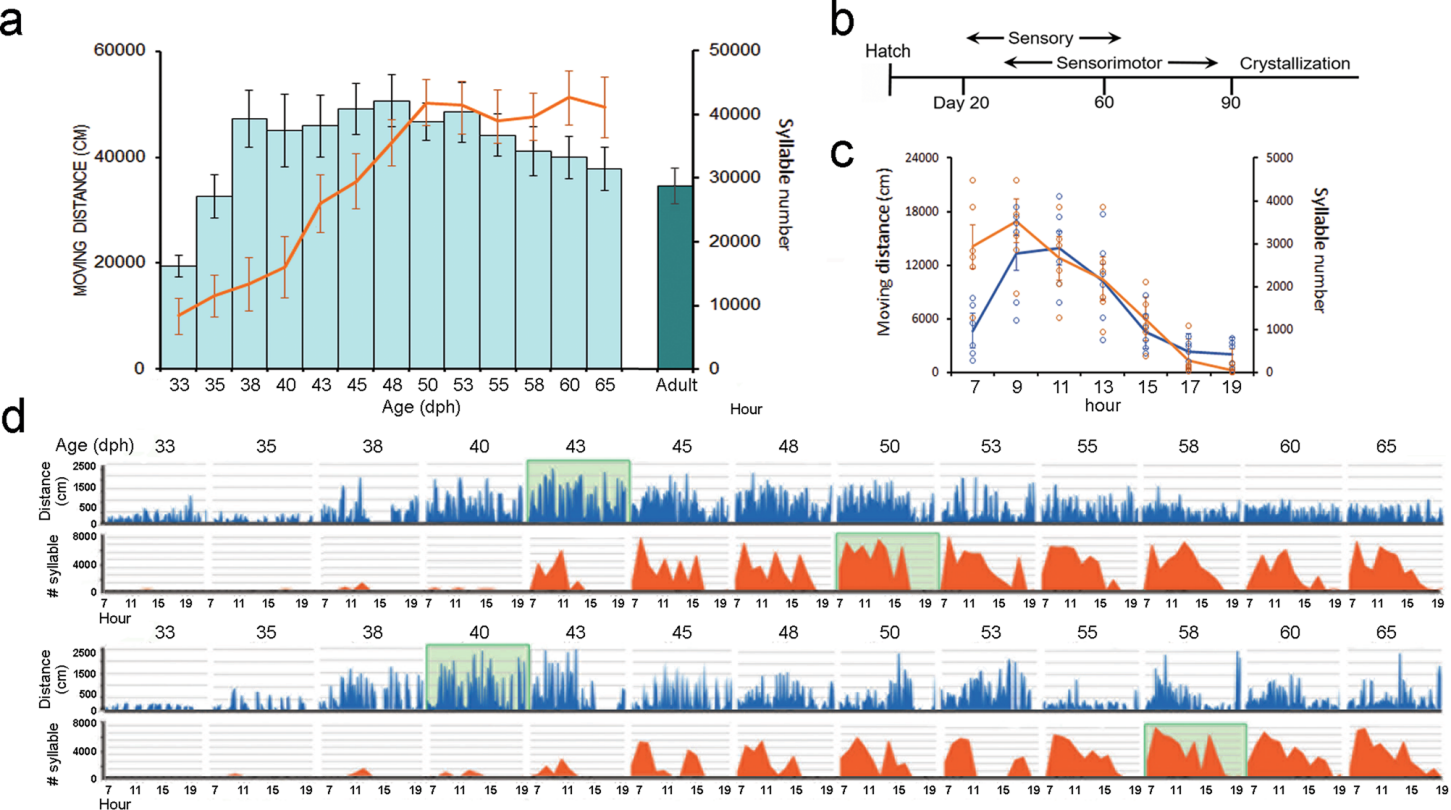
Developmental trajectories of juvenile locomotor movement are associated with vocal practice. **a** The amount of locomotor activity of juvenile male finches (i.e., mean and standard error of total moving distance, shown in light green bars, *n* = 13 birds) across part of the sensitive period of vocal learning from 33-65 dph. Each juvenile was housed in a semi-social environment. Each blue dot depicts the mean moving distance of each individual bird at a given age. The adult males (dark blue bar, 183–390 dph, *n* = 10 birds) had less locomotor movement than that of juveniles. The peak of juvenile locomotor activity (around day 38) came before the peak of juvenile song production (after day 50; orange line depicts the mean of the total number of syllables produced from 33-65 dph). **b** Developmental schedule of vocal learning in zebra finches^12^. **c** The within-day motor activity of juvenile locomotor movement (blue line represents the mean of the total distance moved) and number of song syllables produced (orange line). Each data point is the mean of total moving distance (cm) or total syllable number throughout the day, across the sensitive period from 33 to 65 dph for 6 juvenile males. **d** Examples show two juveniles’ developmental trajectories of locomotor activity from 33 to 65 dph. Top panel of each bird: each blue line represents the total moving distance (cm) per 6 min; bottom panel: the orange graph represents mean number of song syllables per hour. The light green block depicts the peak of the locomotor activity or song production in each bird.

To identify the developmental association between juvenile locomotion and vocal learning, we closely monitored juvenile locomotor movement and song production during the sensitive period of song learning (Fig.1a, b). Juvenile zebra finches fledged at around 18-20 days posthatching (dph), and started exploring their surroundings by hopping and learning to fly. The amount of locomotor movement slowly increased after fledging and reached a peak at around 38-42 dph (Fig. 1a, d, Supplementary Movie 1).

**Fig. 2:**
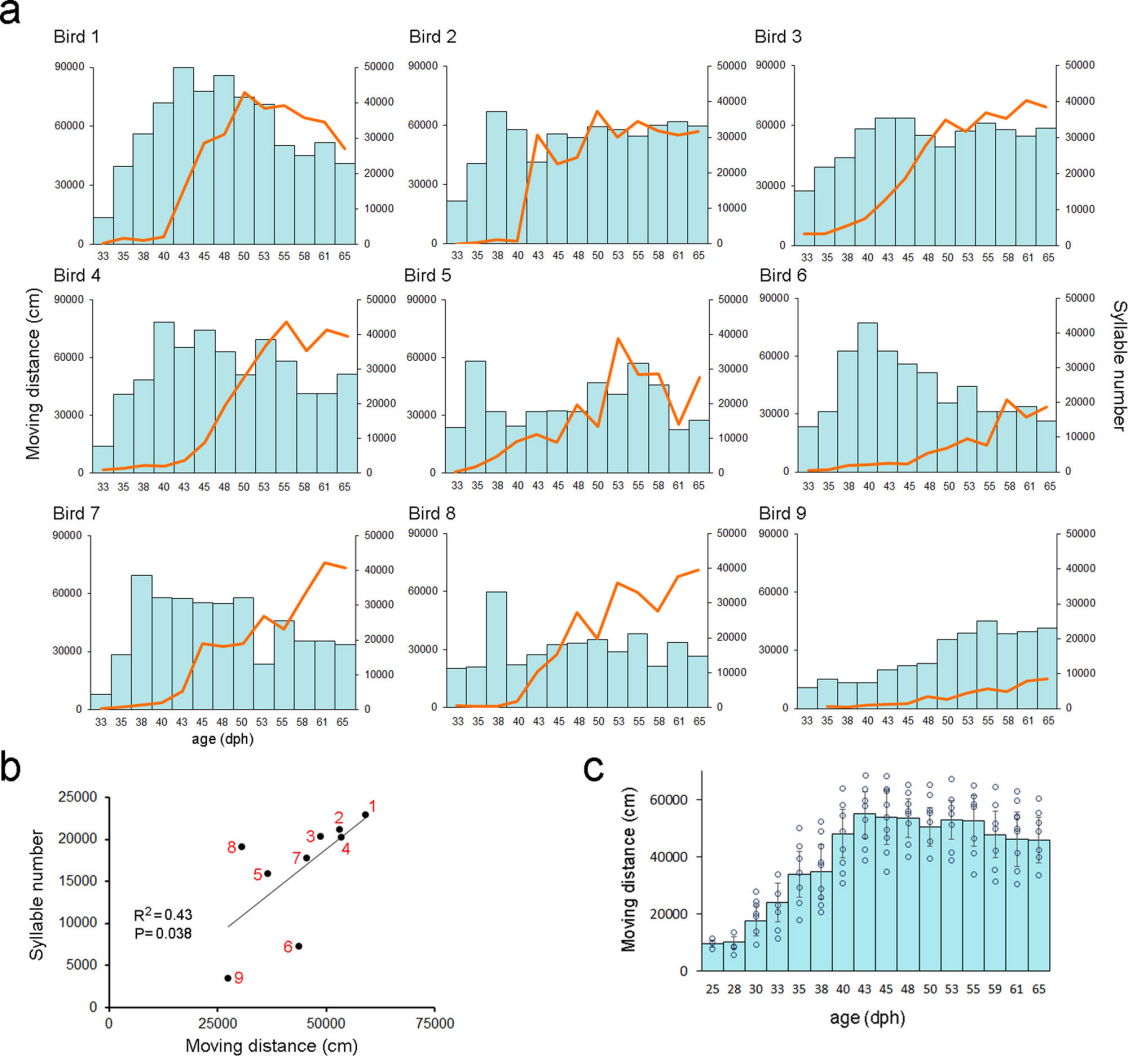
Developmental trajectories of individual juveniles’ locomotor movement and song production. **a** Each graph shows the trajectory of locomotor activity (light blue bars) and syllable production (orange line) of each juvenile male (*n* = 9 birds that had complete song recordings) across part of the sensitive period of vocal learning from 33 to 65 dph. The three birds on the top (Birds 1-3) had more locomotor movement, more song syllable production, and better tutor song imitation (87, 89, and 86% of similarity match to the tutor song respectively), whereas the three birds on the bottom (Birds 7–9) had less movement, less song production and poor tutor imitation (45, 47, 43%, respectively). These juveniles were housed in a semi-social setting in Fig. 1a. **b** The amount of locomotor activity (average of cumulative moving distance from 33-65 dph) is correlated with the production of syllable numbers. Each number denotes a bird ID from (**a**). **c** Developmental trajectories of juvenile locomotor movement in a social setting. The amount of locomotor activity of juvenile male finches (i.e., mean and standard error of cumulative moving distance, shown in light blue bars, *n* = 6 birds) across part of the sensitive period of vocal learning from 33 to 65 dph. Each juvenile was housed together with its father tutor from 0 to 65 dph. This social setting, however, did not allow us to quantify the song development.

The surge in the developmental trajectory of juvenile locomotor activity occurred universally, when juveniles were housed in a semi-social environment (that is, each juvenile was housed together with its father tutor for two consecutive days, alternated by being housed alone for two consecutive days. Housing the juvenile alone allowed us to simultaneously record song development and movement trajectory, *n* = 13 birds, Fig. 1a). Similar movement trajectory was also observed when a juvenile was socially housed together with a father tutor from 0 to 65 dph **(**Fig. 2c; *n* = 6 juveniles**)**, suggesting the juvenile’s surged movement was not due to social isolation. In both settings, juveniles had a surge of locomotor movement at around 38–42 days of age. Compared to the juvenile, adult males (*n* = 10 birds, approximately 185-390 days of age) had less movement than juveniles recorded from 33 to 65 days of age (the average of daily cumulative movement for adult males: 34527.5 ± 3291.8 cm, mean ± SEM, for juveniles: 43163.7 ± 3667.4 cm; Mann-Whitney U test, U = 29, *P* = 0.047, Fig. 1a).

The escalating juvenile movement occurred during the sensitive period of vocal learning in zebra finches (Fig. 1a), when juveniles produced “babbling” subsong and developed forebrain song circuits^12,16,17^. The surge of locomotor movement occurred days (11.5 ± 1.69 days) before the surge of plastic song production at around 50 dph^18–20^ (Figs. 1a, d and 2; the plastic song was defined as the song rendition with recognizable syllables). Total locomotion and syllable production from 33 to 65 dph increased in the morning and then declined in the afternoon (Fig. 1c). Moreover, the individual variability of moving distance was correlated with the production of syllable numbers, as individual juveniles who had more locomotor activity tended to produce more song syllables (Pearson’s correlation coefficient *R*^2^ = 0.43; *P* = 0.038, n = 9 birds, Fig. 2b).

**Fig. 3:**
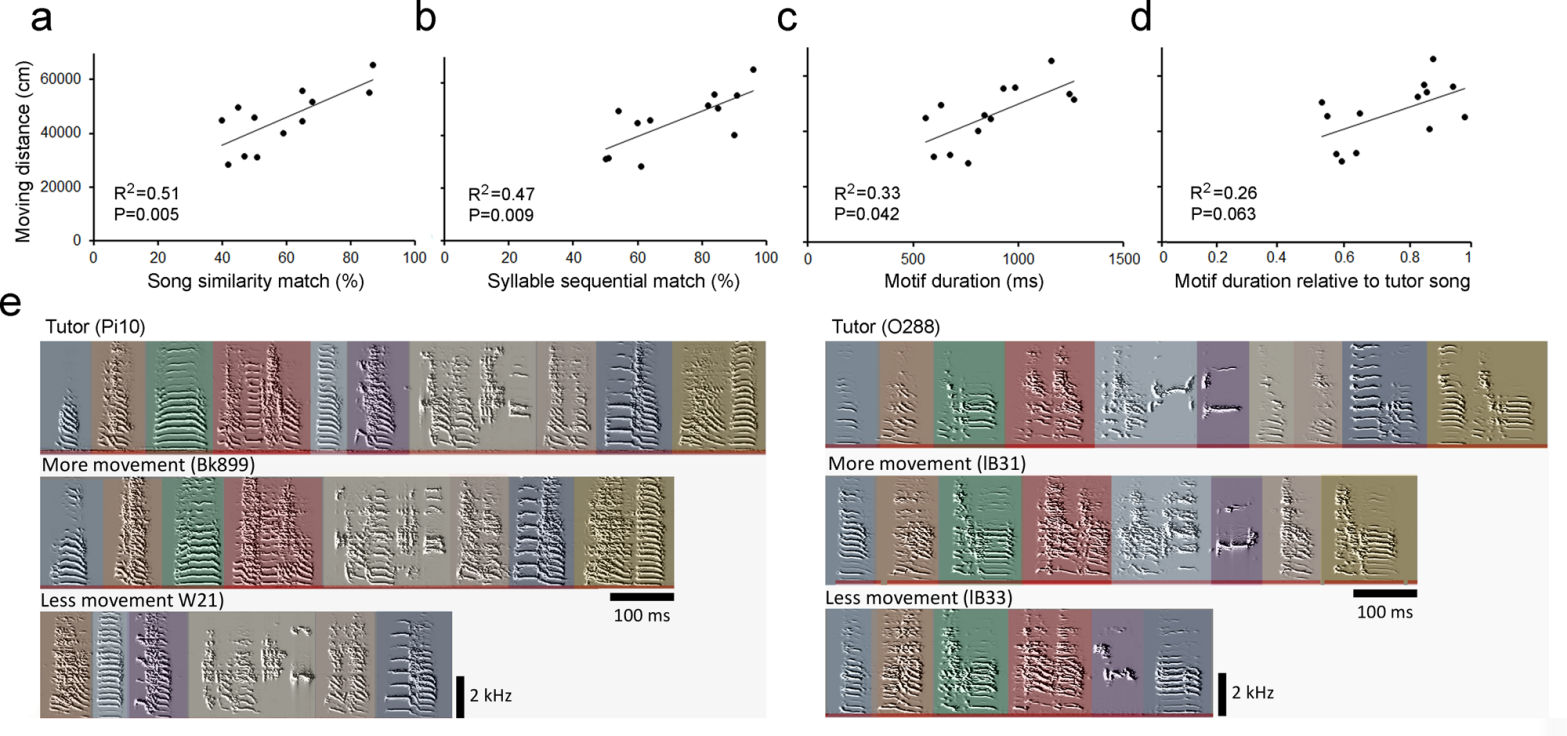
Individual variation in juvenile locomotor activity was correlated with precision of tutor song imitation. **a** The average amount of locomotor activity (i.e., mean of total moving distance from 35 to 65 dph, *n* = 13 birds) of individual juveniles was positively correlated with similarity match of tutor song. **b** The syllable sequential match (%) of the tutor song was positively correlated with the amount of locomotor activity. **c** The duration of multisyllable motif was positively correlated with the amount of locomotor activity, and similarly, **d** however, individual’s locomotor movement was not associated with the ratio between the tutee’s motif duration and tutor’s motif duration, that is, the ratioi between the tutee’s motif duration and tutor’s motif duration. **e** Examples show the correlation between juvenile locomotor activity and song learning. The juveniles (Bk899 and lB31) that had more locomotor activity at the juvenile stage also produced a better match of tutor song (60% and 65% respectively) and sequential match (81% and 84%, respectively) than their biologically-related siblings (W21 and lB33; 44% and 45% of tutor song similarity match and 58% and 53% sequential match). Both father tutors (Pi10 and O288) had long motif duration (1448 and 1103 milliseconds respectively). Sonograms depict the crystallized songs recorded from sibling males at around 100 dph, each semi-transparent colored bar represents a syllable.

Importantly, individual variability of juvenile locomotor activity was associated with the capability of song imitation (Figs. 2 and 3). Juveniles that had more locomotor movement were better able to imitate their tutors, as they had higher similarity match in song syllables (Fig. 3a, Pearson’s correlation: *R*^*2*^ = 0.51, *P* = 0.005), and multi-syllable sequential order (Fig. 3b, *R*^*2*^ = 0.47, *P* = 0.009). An individual’s locomotor movement was also associated with its song motif duration (Fig. 3c, e, *R*^*2*^ = 0.33, *P* = 0.042), but not the ratio to the tutor’s motif duration (that is, the ratio between the tutee’s motif duration and tutor’s motif duration; (Fig. 3d, e; *R*^*2*^ = 0.26; *P* = 0.063). This correlation was greater if the tutor song motif had longer duration (that is, >900 milliseconds, or least 5–6 syllables, see examples in Fig. 3e; *R*^*2*^ = 0.55, *P* = 0.003). Moreover, juveniles that had more locomotor movement and more syllable production also had faster progression of song development (Supplementary Fig. 1).

### Social influence on juvenile locomotor activity

Can escalated juvenile locomotor activity be influenced by social interaction or locomotion of other birds? When two young siblings were housed together with their father tutor, juvenile siblings were more likely to move and rest at approximately the same time, either within a day or throughout the sensitive period (Supplementary Fig. 2). The crystallized songs of these siblings were more similar to each other than that of the adult tutor (*n* = 8 birds, Wilcoxon signed rank paired test, *Z* = −2.02, *P* = 0.037), as shown in a previous study^21^ (Supplementary Fig. 2. Under this social setting, however, we were not able to track the song development trajectory of each sibling.

### Manipulation of juvenile locomotor activity

The robust developmental association between juvenile locomotor practice and vocal learning could be due to **1)** a broader and shared development program that operates independently of the development of locomotor and vocal learning; or **2)** alternatively, an earlier emerging locomotor program that may subsequently promote the development of vocal-motor learning programs. To test the possible effect of locomotor movement on subsequent vocal development and its associated neural plasticity, juvenile flight movement was manipulated during the sensitive period of song learning (20–65 dph): **a)** Locomotor activity was enhanced by housing a juvenile (*n* = 8) in a larger cage (77.5 × 91.5 × 86.5 cm), which allowed juveniles to have more space for movement. Moreover, the locomotion was restricted by housing the juvenile’s sibling in a smaller cage (25.5 × 30.5 × 33 cm; *n* = 8), the housing environments were otherwise identical. Tutors were mostly chosen from adult males that had a longer duration of motif sequence (approximately 1000 ms consisted of >5–6 syllables per motif, see Fig. 3a for examples). **b)** Flight restriction was performed by reversible wing clipping (i.e., cut 6-8 primary feathers at the end of the dorsal major primary coverts, which regrow in a few months; *n* = 8 birds). The wing-clipped birds were unable to sustain upward flight, but could freely hop, glide, fly short distance, and interact with their tutors. To reduce potential stress, wing-clipping was done during the late nestling stage (before fledging), and the bodyweight of wing-clipped birds and intact controls was measured every two weeks.

**Fig. 4:**
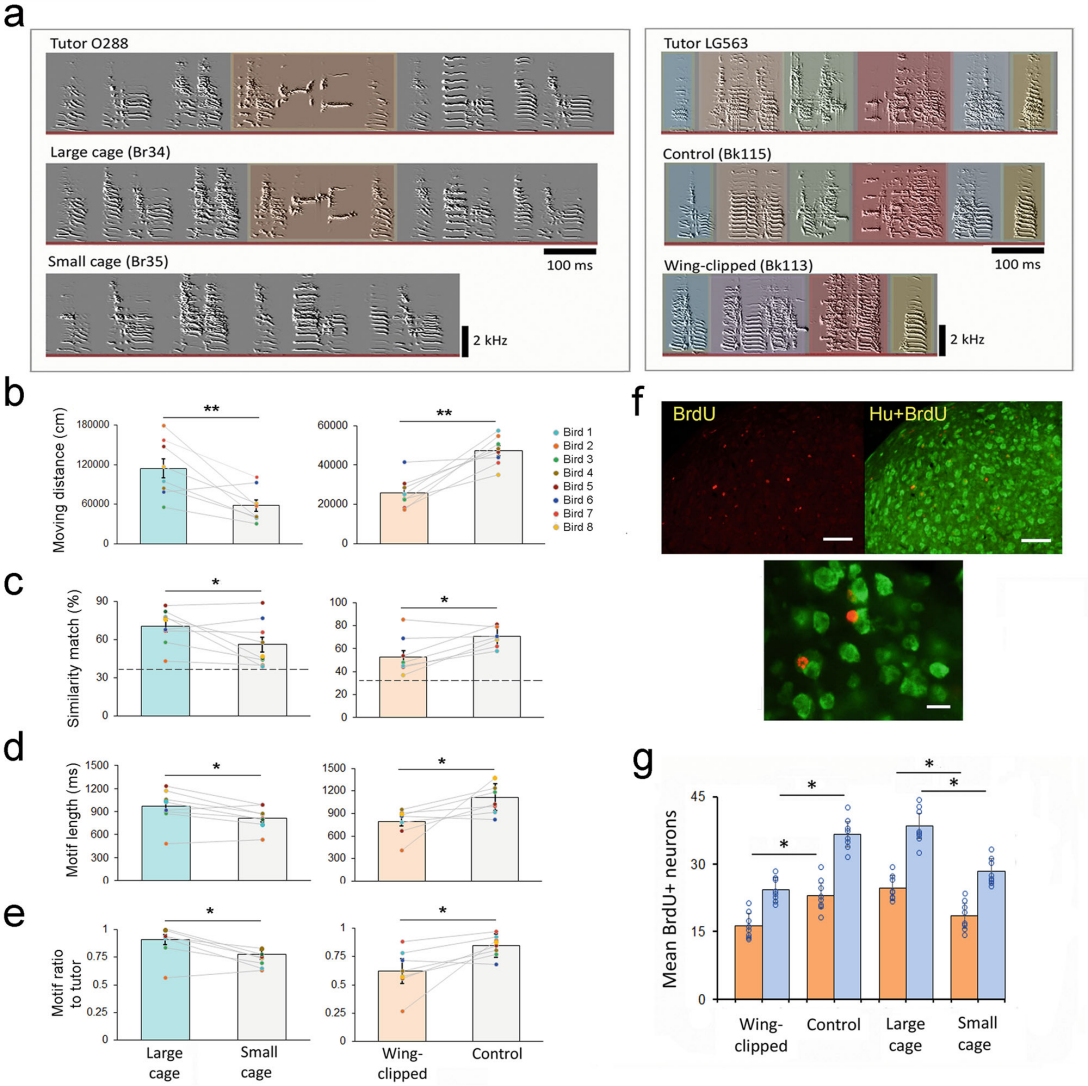
Manipulation of juvenile locomotor activity affected song learning and neural plasticity. **a** Sonograms show two family examples of song learning by either promoting locomotion activity with large cage housing, or restricting locomotion with wing clipping or small cage housing, during the sensitive period of song learning between 30 and 65 dph. Left panel. A juvenile kept in a large cage (Br34) had a longer multi-syllable sequence and better tutor imitation (76% match), compared to its sibling that was housed in a small cage (Br35 with 47% similarity match): the highlighted syllable (shown in Tutor 228 and a juvenile Br34) was a complex syllable and was missing in Br35. Right panel, wing-clipped bird (Bk113) had a shorter motif duration and lower similarity match (45%) compared to its tutor’s song (LG563) or its intact-control sibling (Bk115; 86% similarity match to tutor song). Similar syllable type between the tutor and the tutees was highlighted with the same color, the wing-clipped bird (Bk113) produced fewer syllables and syllables were less similar to its tutor, compared to its sibling (Bk115). **b**–**e** Birds housed in a large cage (green bar, *n* = 8) had significantly more movement (**b**), better song imitation (**c**), longer song motif (**d**), and longer motif duration ratio relative to the tutor song (**e**) compared to their siblings (light gray bar, *n* = 8) that were housed in a small cage (**P < 0.01, *P < 0.05). Similarly, wing-clipped birds (orange bar, *n* = 8) had significantly less moving distance, less similarity match to tutor song, and shorter motif length than their intact-control siblings (**b**–**e**), *n* = 8 birds; from 5 adult tutors). Each colored dot in a graph bar depicts an individual bird, and the gray line connects two sibling birds from the same clutch. The dashed lines in (**c**) represent the average similarity score between adult tutors (*n* = 8) and unrelated juveniles (*n* = 15) from other families. **f** BrdU-labeled neurons in a song nucleus HVC. Red: BrdU-labeled cells, Green: Hu-labeled neurons. Scale bar= 50 µm on the top images and 10 µm on the bottom image. **g** Juveniles housed in a smaller cage (*n* = 8 birds) and wing-clipped birds (*n* = 8 birds) had fewer BrdU-labeled neurons in two forebrain song nuclei (HVC and Area X, light orange and light blue bars respectively; error bars depict the mean and standard error of the number of BrdU+ neurons) compared to the birds housed in a larger cage and intact-control birds. Each dot represents an individual bird.

Compared to the siblings who were housed in a smaller cage (Fig. 4a-e), juveniles housed in a larger cage (*n* = 8 birds) had significantly more locomotor movement (Fig. 4b (Wilcoxon signed-rank paired test, two-tailed, *Z* = −2.54, *P* = 0.013), better song imitation (*Z* = −2.016, *P* = 0.047), longer song motif (*Z* = −2.43, *P* = 0.02), longer motif duration ratio relative to the tutor song (*Z* = −2.43, *P* = 0.02), and they also have more BrdU+ labeled new neurons in the forebrain song nuclei, HVC and Area X (Fig. 4f, g). These song nuclei are part of the forebrain song circuit that is critical for sensorimotor learning of birdsongs. Similar to the results shown in Fig. 3, individual birds that had more locomotor movement (moving distance) also performed better song imitation (Pearson’s correlation coefficient, *R*^*2*^ = 0.32, *P* = 0.04). On the contrary, juveniles housed in a smaller cage where they could freely move around but had limited space to fly could still imitate tutor song, but had shorter motif duration and less precision (see details in Fig. 4b-e, Supplementary Fig. 3). Similarly, flight restriction by wing-clipping was associated with reduced locomotor activity and impaired vocal imitation and forebrain neurogenesis (*P* < 0.05; Fig. 4a-g). Wing-clipped juveniles had significantly less movement (Wilcoxon paired test, *Z* = 2.53, *P* = 0.012), their crystallized song matched the tutor song less (*Z* = 2.42, *P* = 0.015), and had a shorter motif length (*Z* = 2.15, *P* = 0.028), compared to their intact, sibling controls (Fig. 4b-e). The bodyweight of wing-clipped birds was not significantly different from that of intact controls during the age from 35 to 65 dph (12.1 ± 1.3 vs.11.9 ± 1.6 gm; Wilcoxon signed-rank paired test; *Z* = 1.47, *P* = 0.16). Additionally, wing-clipped birds had significantly fewer BrdU+ labeled newborn neurons in two of the cortical-basal ganglia song nuclei, HVC and Area X (Wilcoxon paired test; *Z* = 2.06, *P* = 0.031 in HVC; and *Z* = 2.01, *P* = 0.043 in Area X; Fig. 4f, g).

## Discussion

Our study provides experimental evidence that juveniles developed surged locomotor activity that occurred before the surge of vocal development during the sensitive period of vocal learning. Moreover, individual birds that had more movement tended to develop better song imitation and had longer duration of song motif. The close association between locomotion and vocal-motor learning could be due to a broader neurogenetic program that operates independently on different sensorimotor systems. Alternatively, the earlier surged juvenile locomotion may facilitate the later development of vocal learning or other cognitive function. This hypothesis is supported by our results that the manipulation of locomotor movement by altering cage size or wing-clipping significantly affected vocal learning and neural plasticity (that is, neurogenesis) in the developing song system. The manipulative results are consistent with previous studies that locomotor training or exercise in juvenile rodents may increase hippocampal neurogenesis and gene expression^22,23^. The juvenile’s early developing locomotor program may thus promote, but not be required, for the development of vocal learning program.

It is worth noting that, while experimental manipulation in cage sizes or wing-clipping significantly affected locomotor activity and song learning, these manipulations may also affect the juvenile’s motivation for motor practice. Regardless, we still found a positive correlation between the amount of locomotor movement and precision of song imitation when birds were housed in the same experimental condition. Individuals birds who were wing-clipped or housed in a smaller cage, but were more motivated and had more locomotor activity, tended to have better imitation of a tutor song (Supplementary Fig. 3). These results suggest environment-induced locomotor changes may promote vocal learning, but individual birds also have intrinsic capacity and developmental constraints for locomotor or vocal development^24,25^.

The surged locomotor trajectory is universally developed in juvenile finches, regardless of the housing environment (social or semi-social environment, Fig. 1a and Supplementary Fig. 1). This surged locomotor movement in juveniles is therefore less likely to be induced by stress, and is reminiscent of the intrinsic developmental trajectory of vocal learning. Why do juvenile songbirds develop age-sensitive locomotor activity? As escalating vocal practice is critical for birdsong development, development for flight or other locomotion might be essential motor practice necessary for later fine locomotor control, such as long-distance flight dispersal or maneuver in wild zebra finches^26^.

When juvenile songbirds first come out of their nest, they instinctively learn to fly, explore their surroundings, and listen to and imprint on their father’s song^27^. A few weeks later, when developing babbling subsong, they have a surge in flight and locomotor activity, followed by escalating production of the plastic song. Presumably, we speculate that the locomotor program and its developmental transitions may induce substantial gene expression changes and modulate multiple neuromuscular systems for breathing, rhythmic control, sensory perception, circuit formation, and sensorimotor coordination^28–30^. These changes may affect later-developing vocal motor programs to generate and fine-tune imitated syllable structures and multi-syllable sequences^31^. For example, in humans, increased locomotor activity during child development affects substantial epigenetic and structure changes in multiple brain regions that are keyed to the development of other sensorimotor learning and cognitive processes^32,33^. It would be interesting to see whether the locomotor activity can also affect other singing-related body gestures (such as the courtship dance) which might require motor learning from the tutor as well^34,35^.

Our study may shed light on the development and evolution of fine motor learning skills. For example, the early development of locomotor or gesture learning, shaped by both genetic and environmental interaction, may have a profound influence on later development of language or speech learning. Vocal learning songbirds can thus provide a suitable animal model to identify and manipulate the neurogenetic substrates of the interconnections between developing locomotion and developing fine motor learning skills.

## Methods

### Movement tracking

To identify and detect the locomotor movement, each bird had the crown feathers (on the top of the head) painted with non-toxic water-based acrylic paint (Wildfire Luminescent Paint, Modern Masters, Supplementary Movie 1). To avoid detection errors of color coding, we used either blue or green colors for individual coding in juveniles because the preliminary test showed these two color codes provide the most reliable marker for color detection and quantification. The wire cages had the top wires removed and replaced with plexiglass for video recordings. Each cage was set up with two wooden perches that ran the width of the cage and were approximately 35.2 cm apart from each other, along with a small center perch/ cuttlebone and a feeding station on the front and center floor of the cage. Videos were recorded using network cameras (Model DCS-942lB1, D-Link Corporation) mounted above the center of each cage and covered most of the field of the cage. The videos were recorded during the entirety of the light part of their day:night cycle (12 L:12D). The 12 h (0700-1900) of video each day were broken up into 6 min trials (that is, a total of 120 6-minute time bins per day). Pearson’s correlation coefficient was used to examine the relationships between percent song similarity to tutor and movement across individuals.

#### Semi-social setting (n = 12 birds)

We tracked locomotor activity of juvenile male zebra finches during the sensitive period of song learning, 33-65 dph, in a semi-social environment. Semi-social groups allowed us to record and quantify movement and song development simultaneously. Twelve juvenile males were reared by their parents until 30 dph, when most of them became independent. Each juvenile male and its father tutor were then housed in the same cage (standard size: 60 × 40 × 50 cm), separated from other conspecific birds for audio-video recording of song development and tracking of locomotor activity. Locomotor activity was recorded for two consecutive days, alternating with two consecutive days of social interaction with the tutor (by placing the juvenile with its tutor in the same cage) from 33-65 dph.

#### Social setting (n = 6 birds)

To determine if the juvenile movement trajectory observed in the semi-social group is also presented in a social setting, we kept a juvenile with its father tutor (*n* = 6 pairs) together and continuously recorded the juvenile’s locomotor movement from 25 to 65 dph. This social setting allowed us to record movement under one father-one son social setting, but did not allow us to quantify the juvenile’s song development.

#### Sibling effect (n = 8 birds)

To measure the locomotor activity of sibling birds, the locomotor activity of 8 juvenile males from 4 clutches was recorded in a social setting where two male siblings were housed together with their parents in a standard medium-sized cage (see above). Juvenile males (two siblings per clutch) were kept together with their parents during the sensitive period of song learning, from 0 to 65 dph. Two male siblings in each clutch had the crown feathers painted with green and blue color respectively, so that we would be able to distinguish between each individual for movement tracking.

#### Adult males (n = 10 birds)

To record and quantify the locomotor movement of adult males, each of 10 adult male finches was housed singly in a semisocial cage, where they stayed alone for video recording of movement tracking for two consecutive days, alternating with two days of social interaction with other adult males. Each male was video recorded for 10–12 days.

#### Quantification of locomotor movement

Locomotor activity was acquired and quantified using EthoVision XT14 (Noldus Information Technology) and a custom template was used to create an arena with zones for each perch and feeding area. Arena measurements were calibrated by the program using the 35.2 cm space between perches as a guide. The detection settings were set so that each subject was consistently and accurately detected by the program at a rate of 0.99 samples per second. All trial data was exported for data analysis.

To make sure the movement of each experimental animal was consistently and accurately detected by the software, two students were assigned to blindly quantify the movement trajectory of the same bird during the entire recording period (33–65 dph). Each student made sure the color marker of each bird was detected when a bird was located at the four corners of a cage, on the perch, or on the bottom of the cage. The independently quantified scores performed by two students were then compared and the differences in the quantification (moving distance) per day between two scorers was less than 5%. This research has been approved by IACUC committee at Colgate University.

### Cage size manipulation

Eight clutches of zebra finches with at least two biologically related male siblings per clutch were used. This was to control for any genetic constraints that could potentially impact vocal learning or juvenile neurogenesis. The siblings were housed with their mother and father tutor until approximately 30 dph (range of 30–33 dph). Once they were independent, the two male siblings were removed from the clutch, and one was placed in a large cage (77.5 × 91.5 × 86.5 cm), whilst the other sibling was placed in a smaller cage (25.5 × 30.5 × 33 cm). The father tutor was housed together with one male sibling for two consecutive days and then was transferred to the other sibling’s cage for two consecutive days. The movement was recorded when the juvenile was housed alone without the father tutor.

### Wing-clipping experiment

Wing-clipped experiments were conducted by using 8 zebra finch clutches as each clutch had at least 2 male siblings. One of the siblings from each clutch had 6-8 primary feathers cut on both wings at 15–18 dph before they fledged and started flight movement (to reduce the wing-clipping induced stress). Wing-clipping is a typical veterinarian procedure and causes no bleeding. Wing-clipping did not prevent flight completely but the bird was unable to achieve or sustain upward flight. Birds could still flap their wings with short-distance flight, and freely move and hop around the cage. The wing-clipped males (*n* = 8 birds) were separated from their mothers and female siblings at 28-30 dph and stayed with the father tutors in a medium-sized cage for two consecutive days and then the father was transferred to its sibling (intact control bird) cage for two consecutive days. The locomotor activities were recorded from 31 to 65 dph. All of the birds had their songs recorded at 100–110 dph, so the crystallized song could be analyzed. For the intact control group, the other male sibling (*n* = 8) was kept with their father tutor for two consecutive days, without wing-clipping, in a cage during the same period of recording (31-65 dph).

### Tracking of song development and analysis of song

To record the amount of song syllables produced during part of the sensitive period of song learning between 33 and 65 dph, all of the experimental birds were each kept in a single cage following the movement tracking schedule (see above). A condenser microphone (Audio Technica, AT801) was placed near the center of a cage, and the song development was recorded continuously with Sound analysis Pro (SAP 2011). To analyze the number of song syllables produced, syllables were automatically segmented with a 5 ms silent interval and 22 dB (amplitude threshold to detect a syllable), the raw data was then exported to Excel for quantification of the total count of syllables. For analysis of crystallized song, the songs of focal birds at approximately 100–110 dph were recorded in individual soundproof chambers using SAP 2011^36^.

Motif duration of the tutor song and juvenile song was measured blindly and independently by two scorers. Song motif was defined here as a multi-syllable sequence that was repeatedly presented in a song rendition. Because each bird’s motifs can be variable within or among song rendition, with respect to the presence of some notes, two scorers independently and blindly examined 10 song files (which included at least 10 song renditions) for each bird and chose and measured the duration of the longest repeated motif unit by excluding the introductory notes.

To blindly measure the song similarity score between tutor and tutee, two scorers were each assigned to ten of each individual tutee’s song motifs (without knowing the bird was from what experimental groups), which were compared to ten of their father tutor’s song motifs by using segmented comparisons with a cross correlation setting of asymmetric and time courses function from SAP 2011. The quantification of the sequential match of song syllables was to measure how the syllable sequence in a selected motif matched (%) between the tutor and tutee, a function from SAP.

### BrdU injection

BrdU injections started at 33–35 dph for three consecutive days. BrdU (5-bromo-2-deoxyuridine) (100 µl of 10 mg/ml in sterile water) was injected intramuscularly twice a day. The birds were then released in a standard, medium-sized cage to record the locomotor movement. Between 96 and 103 dph, birds were anesthetized and perfused with 4% PFA. Brains were extracted and postfixed with PFA, then cryoprotected through increasing concentrations of sucrose in PBS (5, 15, and 30%). Brains were then embedded in Neg-50 and stored at −80C. Brains were coronally sectioned at 30 µm in a frozen cryostat.

### Immunohistochemistry and quantification

We followed and modified the protocol from two previous studies^37,38^. In brief, frozen sectioned slides were placed in TBS (pH 7.5) for 5 min, then incubated in 60% formamide in SSC (saline sodium citrate buffer) for 15 min at 55 °C. Slides were washed in SSC at RT for 10 min. They were then incubated in 2 N HCl for 30 min at 37 °C. Slides were placed in sodium borate solution (pH 8.5) for 10 min at RT. Slides were washed 3 times in TBST (0.1% Triton X-100) for 5 min. Blocking solution (Triton X in PBS with 10% goat serum) was then placed on the slides for 30 min at RT. Primary mouse anti-Hu antibody (anti-HuC/D 16A11, Invitrogen) was put in blocking solution at 1:100 and left on slides for 2 nights at 4 C. On day 3, slides were washed 3 times in TBST. Secondary goat anti-mouse Alexa 488 (IgG (H + L) A11001, Invitrogen) was placed in a Triton X in TBS solution at 1:500 on the slides for 2 h. Slides were washed 3 times with TBS. Slides were blocked for 30 min. Primary rat anti-BrdU (rat monoclonal, ab6326, Abcam) in 1:500 in blocking solutions was added to the slides and left at 4 °C overnight. On day 4, slides were washed 3 times in TBS. Secondary goat anti-rat Alexa 555 (A21434, Invitrogen) was in a Triton X in TBS solution at 1:300 and placed on slides for 2 h. Slides were washed 3 times in TBS and coverslipped with aquamount.

Images of the forebrain song nuclei, HVC and Area X, were taken with Leica fluorescent microscope (DMi8) at 63× magnification. Quantification of BrdU-labeled neurons was performed blinded as the bird ID for each slide was covered. Four to six images of each section’s hemisphere (from both hemispheres) were taken of both Hu and BrdU. The Hu and BrdU images of the same area were colocalized in Leica X to identify BrdU labeled newborn neurons. The number of BrdU-positive cells were counted if the BrdU labeled nucleus overlapped with the Hu labeled neurons. These neurons were counted if they were within an identified region of the forebrain song nuclei, HVC and Area X. These were then averaged together to get means and standard errors for different experimental groups for the entire brain. Statistical analysis of Wilcoxon signed-rank paired test and One-way ANOVA with Tukey’s post hoc test were performed.

### Statistics and Reproducibility

All song data collected with Sound Analysis Pro and all trial data from EthoVision XT14 were independently analyzed using SPSS statistics 26.0 (IBM) and JMP 9.0 (SAS institute). One-way ANOVAs with Tukey post hoc test and Wilcoxon signed-rank paired test were used to determine statistical significance in song similarity, song motif length, and BrdU+ labeled neurons among control, wing-clipped, and cage-size birds. For the movement tracking data, three separate one-way ANOVAs with Tukey post hoc tests were performed for each cage to determine the statistical significance of mean distance moved per trial in cm across the four conditions: father, control, cage size group, and/or wing-clipped group. Mann-Whitney U test was used to test the significant difference in locomotor movement between juvenile and adult finches. Each individual animal or behavior was displayed as an individual point in the bar graph with mean and standard error. Detailed statistical methods in each experiment are described in the relevant methods sections and figure legend. Reproducibility can be accomplished by following the protocols or experimental methods mentioned in the relevant method sections.

### Reporting summary

Further information on research design is available in the Nature Research Reporting Summary linked to this article.

## Supplementary information

Supplementary Information

Description of Additional Supplementary Files

Supplementary Movie 1

Supplementary Data 1

Supplementary Data 2

Reporting Summary

## Data Availability

The source data underlying main and supplementary figures are presented in Supplementary Data 1, 2, respectively. All other data are available from the corresponding author upon reasonable request
