# Supplementary material for: A juvenile locomotor program promotes vocal learning in zebra finches
Source: Commun Biol. 2022 Jun 10;5:573. doi: 10.1038/s42003-022-03533-3 (PMC9187677; doi:10.1038/s42003-022-03533-3)
Supplement: Supplementary file 1 — Supplementary Information [file 42003_2022_3533_MOESM1_ESM.pdf]

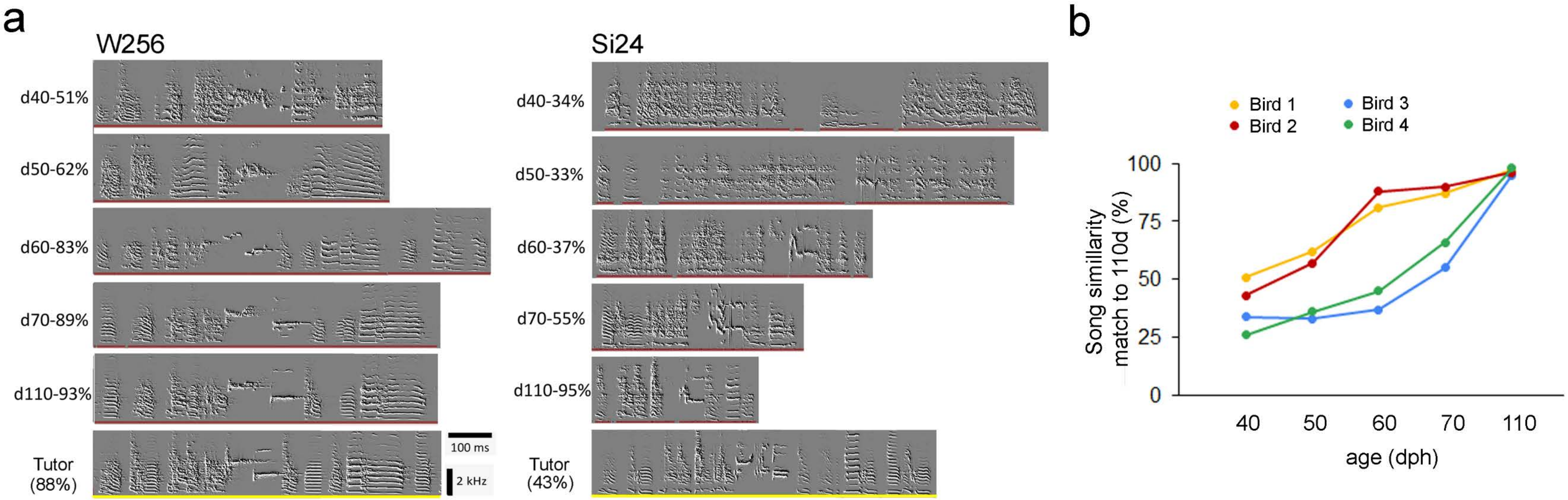

Supplementary Figure 1. Progression of song development varied between individual birds. **a)** Examples show the sonograms of two juveniles during song development from 40-110 dph, Bird W256 had faster progress of song development than Bird Si24 and better tutor song imitation (88% similarity match to the tutor song). W256's plastic song had emerged earlier at d40 (40 days old) with 51% similarity match to his own crystallized song, and the basic song structure had completed by d60 (83% similarity score), and remained stable afterwards. W256 also had more locomotor movement and song production (that is, Bird 3 in **Fig. 2**). Si24, however, had a slower song development, poor tutor imitation (43% tutor match) and less movement (Bird 9 in **Fig. 2**). Both W256 and Si24 seemed to develop a motif strategy of vocal learning[20]. **b)** Examples show the progression of song development of 4 birds from 40-110 days of age. The progression of song development was measured by song similarity match to a bird's own crystallized song at 110 days old. Birds in red and orange lines (Bird 1 and Bird 2) had faster progression of song development than birds in blue and green lines. Each dot depicts the song similarity match between a juvenile's song at different age and its own song at 110 days of age. Blue line was developmental trajectory of Si24, and orange line was W256.

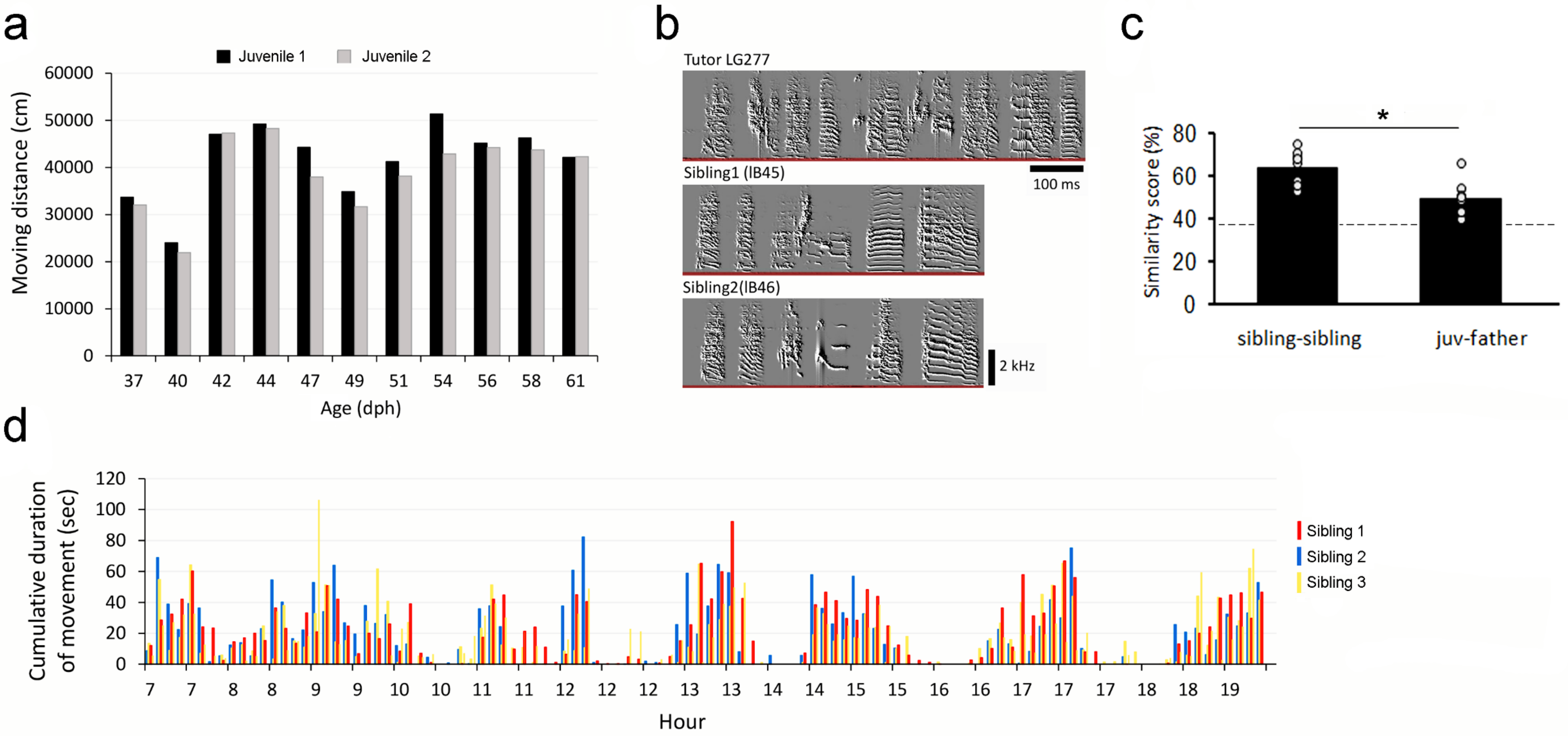

Supplementary Figure 2. Social influence on juvenile locomotor activity and song learning. **a)** An example of two juvenile siblings (represented by black and grey bars respectively) that were housed together with an adult tutor developed a similar movement trajectory from 37-61 dph. **b)** and **c)** Juveniles that were housed together with their siblings developed a crystallized song that was more similar to each other, compared to their adult tutors (n=8 birds; $^*P < 0.05$). The dashed line represents the average similarity score between adult tutors (n=8) and unrelated juveniles from other families (n=20 birds). **d)** Three male siblings at around the same age, 48 dph (the cumulative duration of movement in each of three juvenile siblings was marked with blue, red, or yellow bars), housed together in the same cage, synchronized their daily movement activity from within the day (0700-1900).

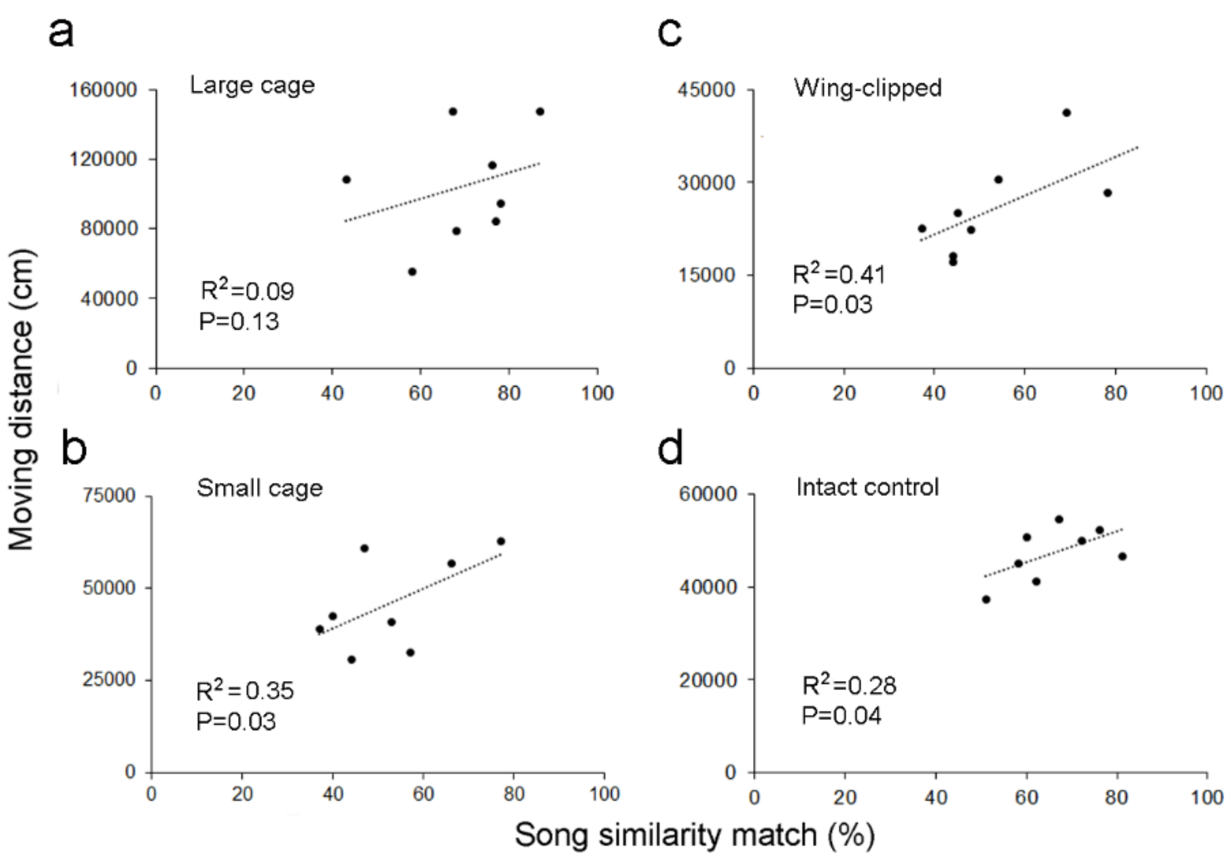

Supplementary Figure 3. When juveniles were wing-clipped (c) or intact control (d), individual birds who had more locomotor activity (total moving distance) tend to have better similarity match to the tutor song. Similar association was also found when juveniles were housed in a smaller cage (a) but not in a large cage (b).
