# Supplementary material for: A juvenile locomotor program promotes vocal learning in zebra finches
Source: Commun Biol. 2022 Jun 10;5:573. doi: 10.1038/s42003-022-03533-3 (PMC9187677; doi:10.1038/s42003-022-03533-3)
Supplement: Supplementary file 2 — Description of Additional Supplementary Files [file 42003_2022_3533_MOESM2_ESM.pdf]

Description of Additional Supplementary Files

File name: Supplementary Data 1
Description: The source data underlying Figures 1-4.

File name: Supplementary Data 2
Description: Source values for Supplementary Figures 1-3.

File name: Supplementary Movie 1
Description: Flight behavior of two juvenile zebra finches. Two juvenile males at 50 dph (each bird marked with blue and green paints respectively) had more flight movement in the morning.
